# Supplementary material for: Hyperoxia causes miR-34a-mediated injury via angiopoietin-1 in neonatal lungs
Source: Nat Commun. 2017 Oct 27;8:1173. doi: 10.1038/s41467-017-01349-y (PMC5660088; doi:10.1038/s41467-017-01349-y)
Supplement: Supplementary file 1 — Supplementary Information [file 41467_2017_1349_MOESM1_ESM.pdf]

SUPPLEMENTAL FIGURES

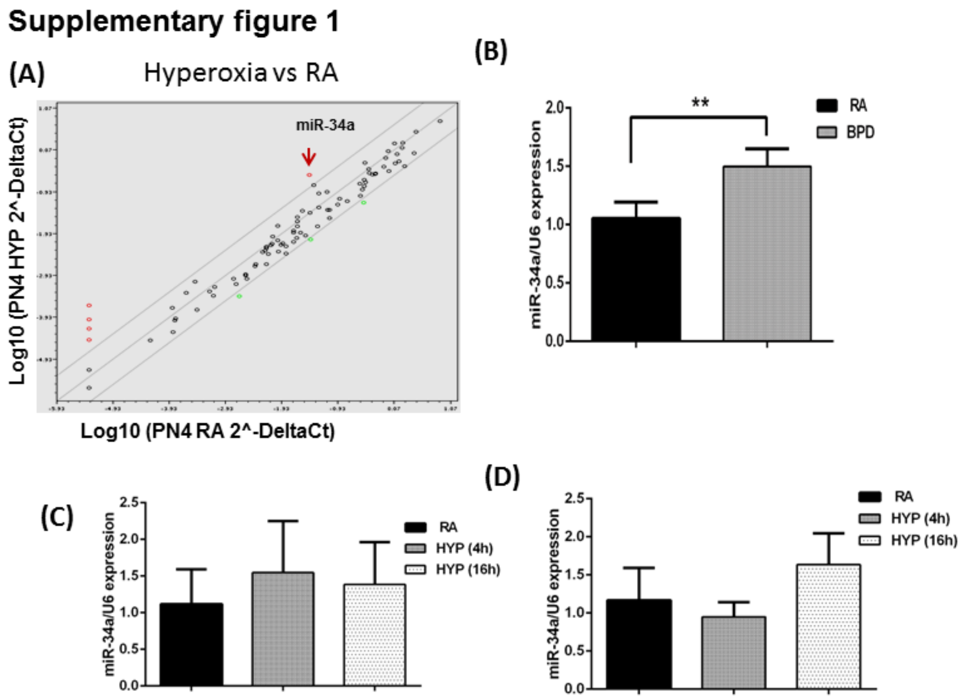

Supplementary Figure 1. (A) Microarray for 80 miRs was performed at PN4 WT RA and HYP mice lungs and Dot plot of LOG10 values were plotted. **(B)** miR-34 expression in newborn mice lungs at PN14 RA WT vs. BPD model. **(C-D)** Mouse lung endothelial cells and macrophages were isolated from newborn mice lungs at PN7 and exposed to hyperoxia (95%) for 4 and 16h. miR-34a expression was performed by real time PCR.WT: wild type; RA: room air; PN: postnatal; HYP: hyperoxia; BPD: bronchopulmonary dysplasia. Values are means ± SEM of a minimum of 3 animals in each group. **$p<0.01$, Student's t test.

Supplementary figure 2

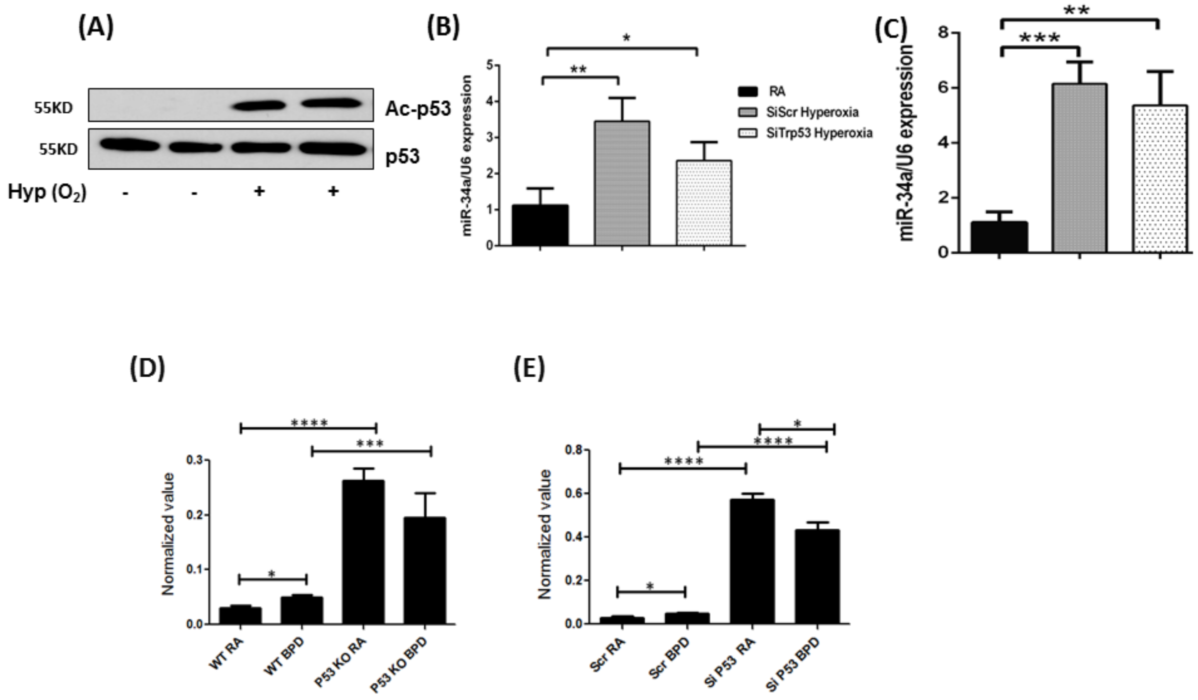

Supplementary Figure 2. (A) RA and hyperoxia exposed MLE12 cell lysates were immunoblotted with acetyl-Trp53 and Trp53 antibodies. **(B)** Silencing of Trp53 by siRNA inhibits hyperoxia-induced miR-34a expression in MLE12 cells. **(C)** Representative graph shows expression of lung miR-34a after intranasal administration of Trp53 siRNA to WT PN4 mice. **(D)** Representative graph shows expression of lung miR-34a in WT mice and p53 null mutant mice in the BPD mice model at PN14. **(E)** Representative graph shows expression of lung miR-34a after intranasal administration of Trp53 siRNA to WT mice in our BPD mice model at PN14. RA: room air; WT; wild type; PN: postnatal; BPD: bronchopulmonary dysplasia. Values are means $\pm$ SEM of a minimum of 4 observations (*in vitro* experiments) or 3-4 animals (*in vivo* experiments) in each group. *p<0.05, **p<0.01, ***p<0.001, ****p<0.0001, compared with controls, 1-way ANOVA.

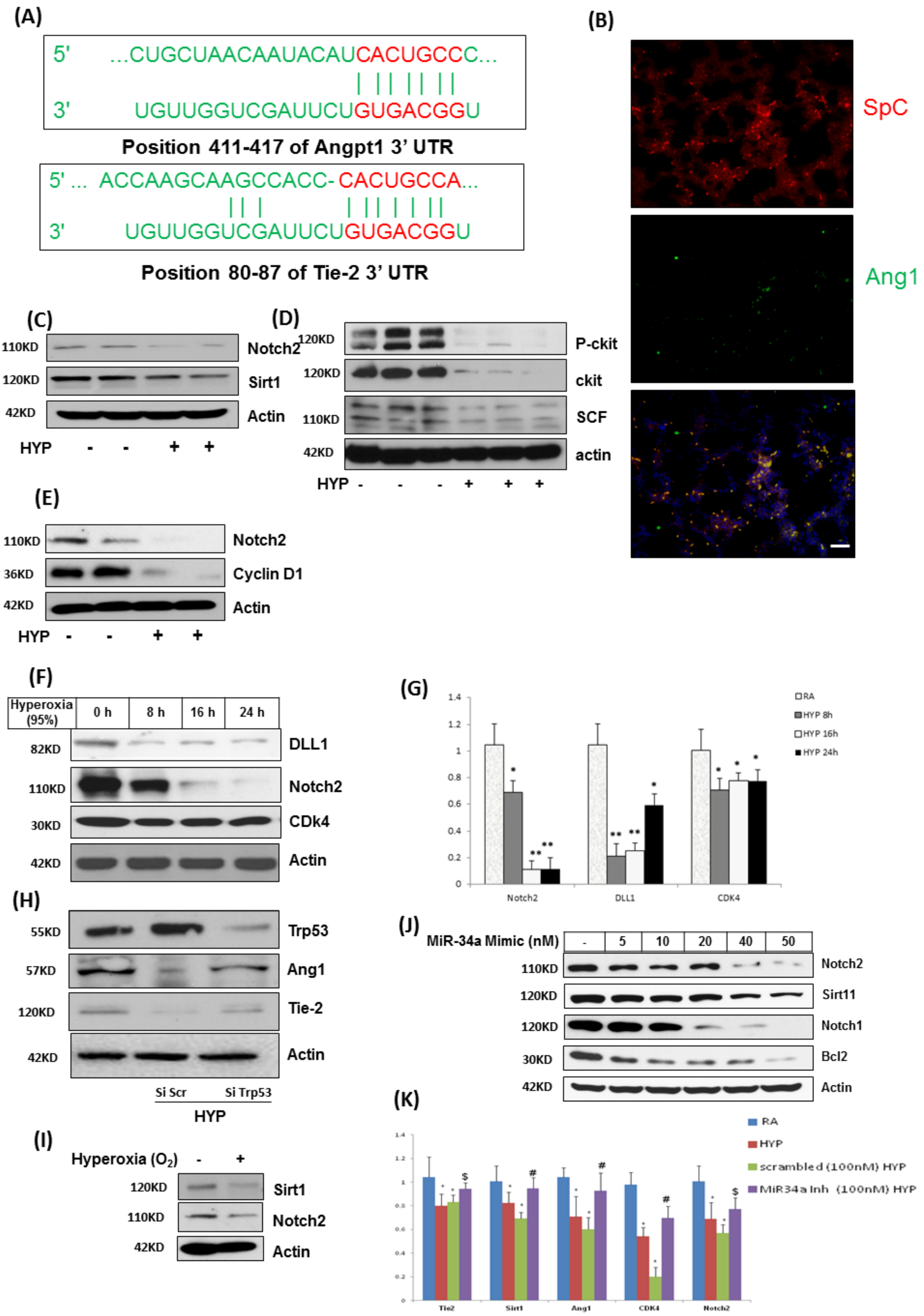
(A)
5' ...CUGCUAACAAUACAUCACUGCCC...
3' UGUUGGUCGAUUCUGUGACGGU
Position 411-417 of Angpt1 3' UTR
5' ... ACCAAGCAAGCCACC- CACUGCCA...
3' UGUUGGUCGAUUCUGUGACGGU
Position 80-87 of Tie-2 3' UTR
(B)
SpC
Ang1
(C)
110KD Notch2
120KD Sirt1
42KD Actin
HYP - - + +
(D)
120KD P-ckit
120KD ckit
110KD SCF
42KD actin
HYP - - - + + +
(E)
110KD Notch2
36KD Cyclin D1
42KD Actin
HYP - - + +
(F)
Hyperoxia (95%) 0 h 8 h 16 h 24 h
82KD DLL1
110KD Notch2
30KD CDk4
42KD Actin
(G)
RA
HYP 8h
HYP 16h
HYP 24h
Notch2
DLL1
CDK4
(H)
55KD Trp53
57KD Ang1
120KD Tie-2
42KD Actin
Si Scr
Si Trp53
HYP
(I)
Hyperoxia (O2) - +
120KD Sirt1
110KD Notch2
42KD Actin
(J)
MiR-34a Mimic (nM) - 5 10 20 40 50
110KD Notch2
120KD Sirt11
120KD Notch1
30KD Bcl2
42KD Actin
(K)
RA
HYP
scrambled (100nM) HYP
MiR34a Inh (100nM) HYP
Tie2
Sirt1
Ang1
CDK4
Notch2

Supplementary Figure 3. **(A)** A sketch of the construction and sequence alignment of the wild-type *Ang1* 3′ UTR (411-417 position; Red letters) and Tie2 (80-87 position; Red letters) 3' UTR with mmu-miR-34a. **(B)** Lung sections from WT mice were either co-immunostained with anti-SpC (red) and anti-Ang1 (green) to confirm Ang1 expression in type2 cells. Scale bar, 10 µM. Images represent 3-4 mice/group. **(C-D)** Western blot showing decreased Notch2, Sirt1, ckit, kit and SCF protein expression in WT hyperoxic lung (PN4) as compared to RA control. **(E-G)** Western blot and densitometry showing decreased Notch2 and cyclinD1 protein expression at 24h of hyperoxia and time dependent decrease in DLL1, Notch2 and cdk4 proteins in MLE12 cells. **(H)** Western blot of Trp53 knockdown MLE-12 cells showed increased expression of Ang1 and Tie2 as compared to hyperoxia exposed scrambled control. **(I)** Type 2 epithelial cell lysate also shows less expression of Sirt1 and Notch2 protein after 4 h of hyperoxia. **(J)** Immunoblotting after transfection of miR-34a mimic to MLE12 cells shows dose dependent decrease in Notch2, Sirt1, Notch1, Bcl2 proteins. **(K)** Densitometry showing increased Tie2, Ang1, Sirt1 Notch2 and cyclinD1 protein expression in miR 34a treated lung lysates as compared to scrambled control after 4 days of hyperoxia. *p<0.05, compared to RA; $p<0.05, compared to HYP groups; #p<0.01, compared to HYP groups.

Ang1: angiopoietin 1; SpC: surfactant protein C; Sirt1: sirtuin1; SCF: stem cell factor; NB: newborn; WT: wild type; RA: room air; HYP: hyperoxia; PN: postnatal. Values are means ± SEM of a minimum of 4 observations (*in vitro* experiments) or 4 animals (*in vivo* experiments) in each group, unless otherwise specified. *p<0.05, **p<0.01, compared with controls, 1-way ANOVA.

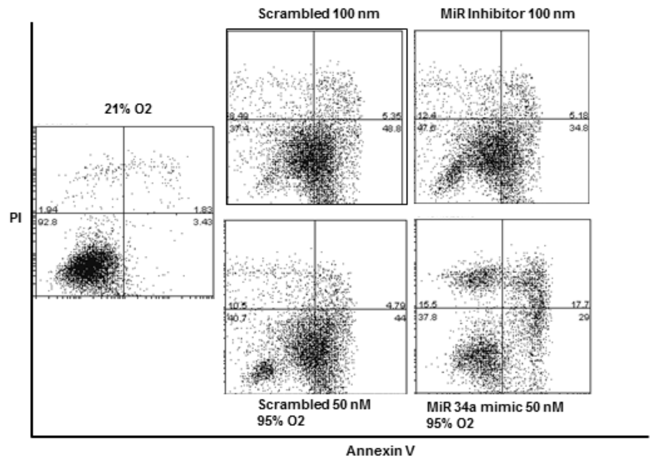

Supplementary Figure 4. MLE12 cells were treated with miR-34a mimic (50 nM) and miR-34a inhibitor (100 nM) along with appropriate scrambled controls and exposed to hyperoxia for 48h, and subjected to Annexin V and Propidium Iodide (PI) assay. Representative FACS scatter plot.

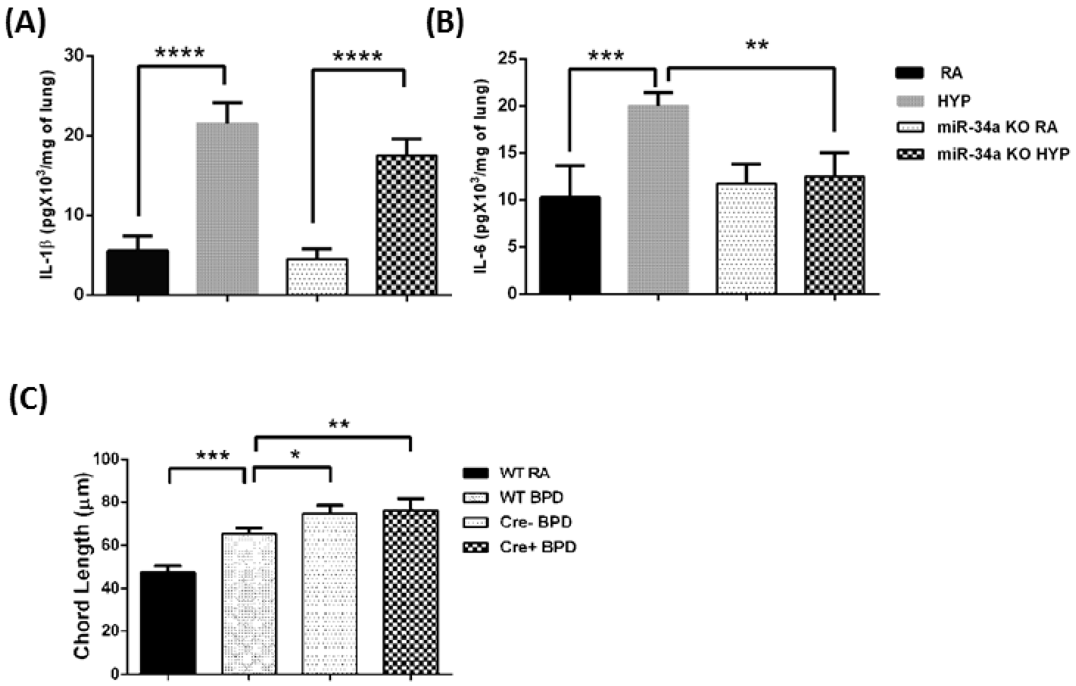

Supplementary Figure 5. (A-B) Bar graphs showing concentration of IL-1β and IL-6 in whole lung lysates of WT and *miR-34a* KO in PN14 RA and BPD mice. **(C)** Graph showing effect of Tamoxifen administration on chord length in different BPD models. WT: wild type; RA: room air; PN: postnatal. IL: interleukin; WT: wild type; PN: postnatal; RA; room air; BPD: bronchopulmonary dysplasia. Values are means ± SEM of a minimum of 4 animals in each group. *p<0.05, **p<0.01, ***p<0.001, ****p<0.0001, compared with controls, 1-way ANOVA.

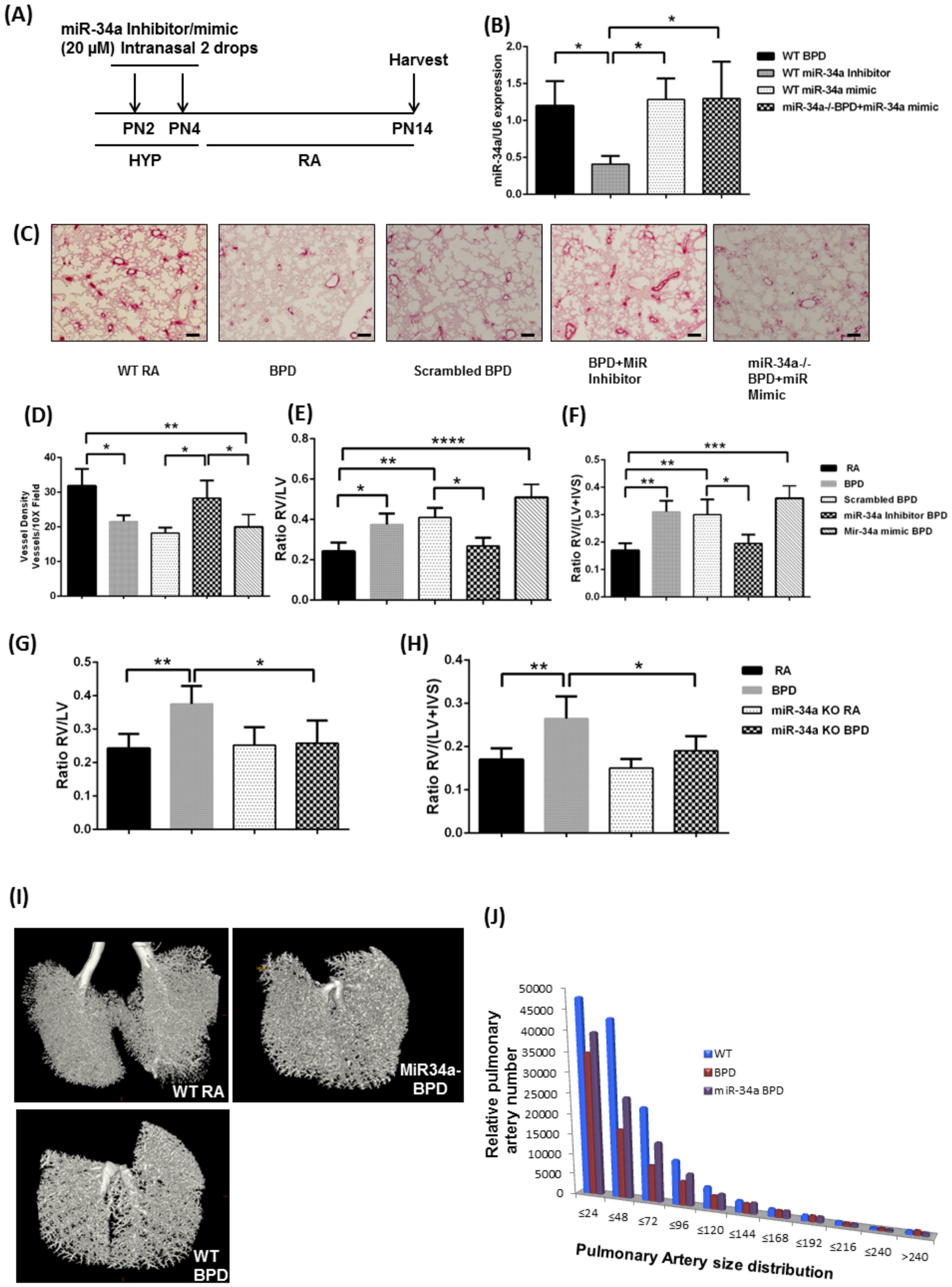
(A)
miR-34a Inhibitor/mimic
(20 μM) Intranasal 2 drops
Harvest
PN2
PN4
PN14
HYP
RA
(B)
miR-34a/U6 expression
WT BPD
WT miR-34a Inhibitor
WT miR-34a mimic
miR-34a-/-BPD+miR-34a mimic
(C)
WT RA
BPD
Scrambled BPD
BPD+MiR Inhibitor
miR-34a-/- BPD+miR Mimic
(D)
Vessel Density Vessels/10X Field
(E)
Ratio RV/LV
(F)
Ratio RV/(LV+IVS)
RA
BPD
Scrambled BPD
miR-34a Inhibitor BPD
Mir-34a mimic BPD
(G)
Ratio RV/LV
(H)
Ratio RV/(LV+IVS)
RA
BPD
miR-34a KO RA
miR-34a KO BPD
(I)
WT RA
MiR34a-BPD
WT BPD
(J)
Relative pulmonary artery number
WT
BPD
miR-34a BPD
≤24
≤48
≤72
≤96
≤120
≤144
≤168
≤192
≤216
≤240
>240
Pulmonary Artery size distribution

Supplementary Figure 6. (A) Schematic representation shows administration of miR-34a inhibitor or mimic at PN day 2 and 4 in the BPD mouse model. **(B)** Bar graph shows lung miR-34a expression after administration of miR-34a inhibitor or mimic in WT mice. **(C)** miR-34a inhibition improves lung angiogenesis in the mouse BPD model.
Lung sections from indicated groups were immunostained with von-Willbebrand Factor (vWF). Scale bar: 100 µm. The mean number of total **(D)** vWF positive vessels per 10X field (means ± SD, n=5 per group) are shown in the bar graphs. **(E-F)** Bar graphs showing the ventricular free wall thickness as indicated by right ventricle (RV) to left ventricle (LV) ratio and RV hypertrophy as indicated by RV/(LV + interventricular septum or IVS) at PN14. **(G-H)** Improved RV to LV ratio and RV/(LV + IVS) were noted in *miR-34a KO* BPD mice as compared to WT controls. **(I-J)** micro-CT displayed numerous small pulmonary arterioles extending into the capillary circulation in WT RA lungs, whereas there were strikingly fewer arterioles and capillaries in lungs from the BPD mouse model; in contrast, the miR-34a KO BPD mouse lung revealed an improved lung vascularization phenotype (n=1, for each). NB: newborn; PN: postnatal; BPD: bronchopulmonary dysplasia; WT: wild type; RA; room air; KO: knockout or null mutant. Values are means ± SEM of a minimum or 4 animals (*in vivo* experiments) in each group, except micro-CT data. *p<0.05, **p<0.01, ***p<0.001, ****p<0.0001, compared with controls; 1-way ANOVA.

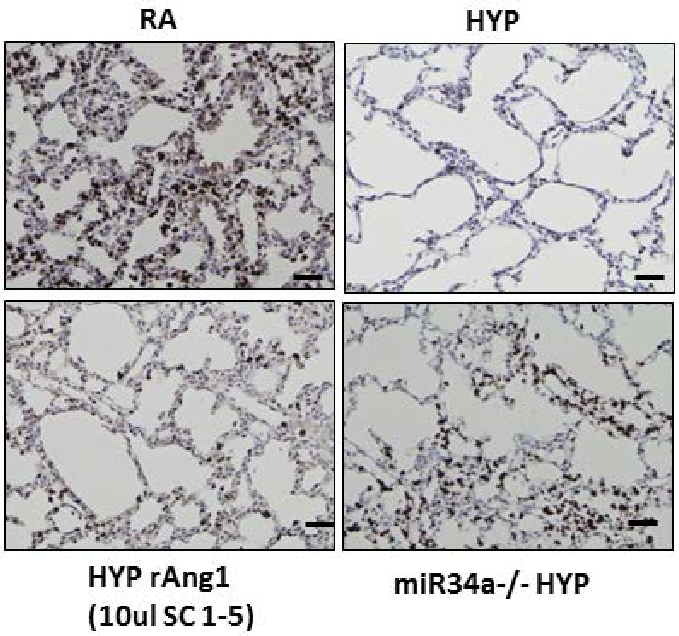

Supplementary Figure 7. Lung immunostaining showing decreased Ki67 in PN4 HYP samples as compared to RA controls, but treatment of Ang1 improved the Ki67 staining. *MiR-34a* KO lung samples also showed increased Ki67 as compared to WT. PN: postnatal; HYP: hyperoxia; RA; room air; Ang1: angiopoietin 1; KO: knockout or null mutant; WT: wild type. A minimum of 4 animals in each group were used.

Supplementary figure 8

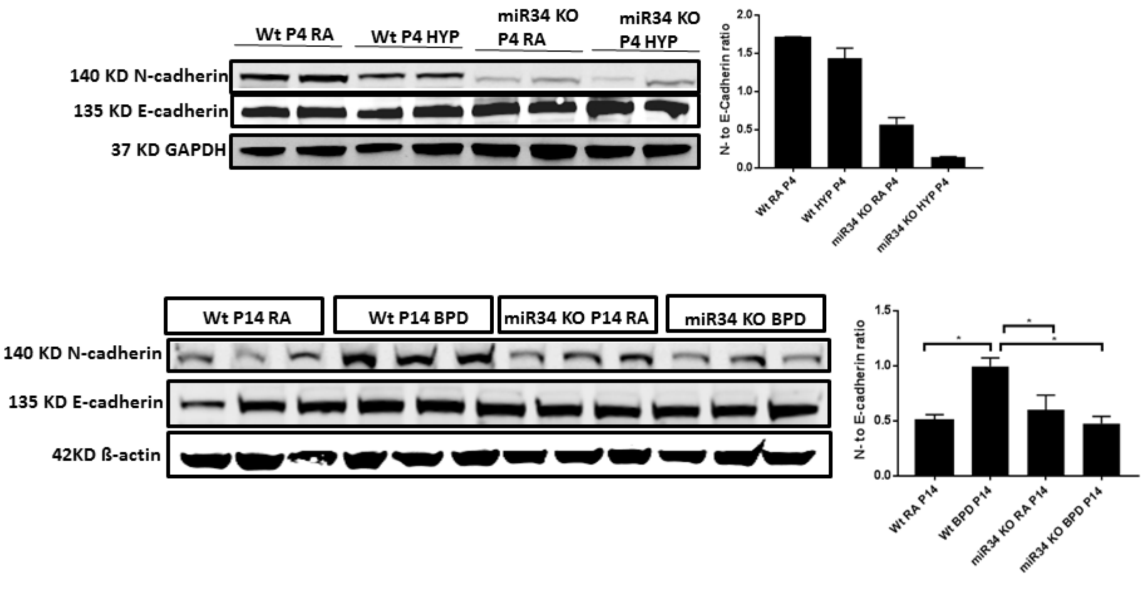

Supplementary Figure 8. Western blot and quantification of the same showing mesenchymal marker N-cadherin is decreased in the miR-34a null mutant mice, as compared to Wt BPD lungs upon hyperoxia exposure at P4 and in the BPD model at P14, with no change in the epithelial marker (E-cadherin). P: postnatal; HYP: hyperoxia; RA; room air; KO: knockout or null mutant; Wt: wild type. Data from 2-3 animals in each group were used. *p<0.05, compared with controls, 1-way ANOVA.

Supplementary figure 9

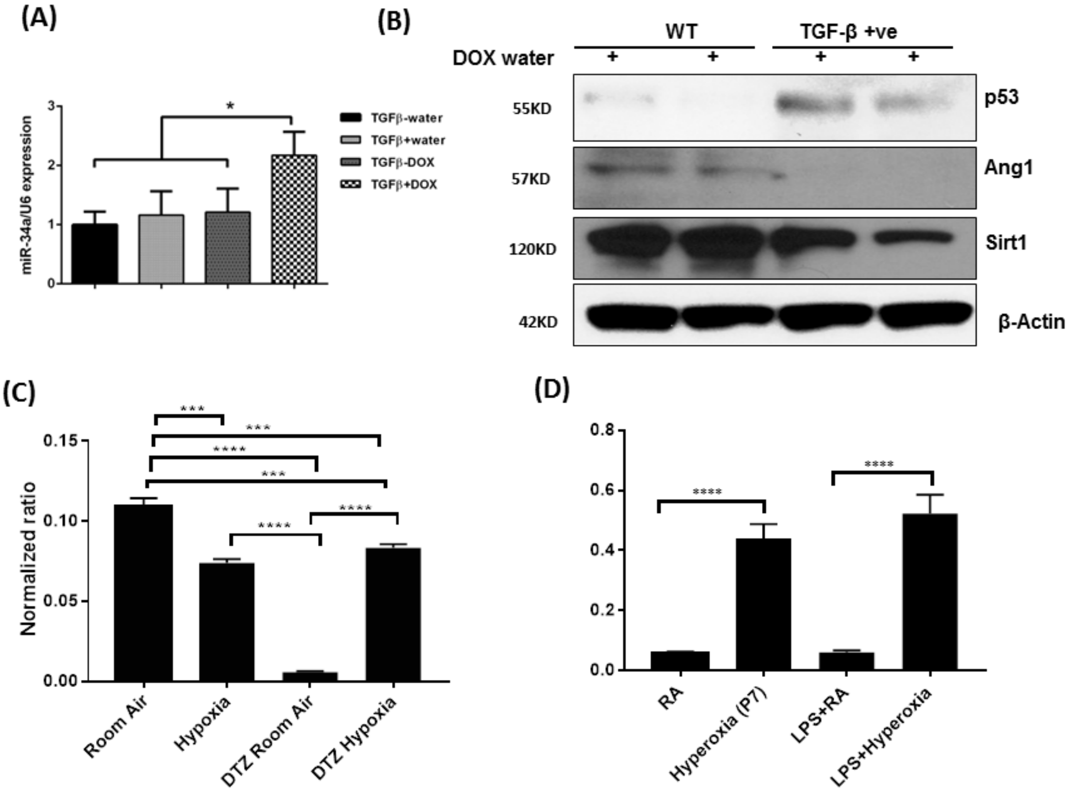

Supplementary Figure 9. Additional mice models for increased miR-34a and decreased Ang1 in BPD. **(A)** Bar graph shows the increased miR-34a expression in TGFβ1 transgenic mice lungs after doxycycline administration from PN7-PN10 at RA. **(B)** Lung lysates of PN10 TGFβ1 positive and controls mice were immunostained with p53, Ang1 and Sirt1 antibodies. **(C)** Bar graph shows decreased miR-34a expression with hypoxia and decreased TGFβ signaling [using inducible dominant-negative mutation of the TGF-beta type II receptor (DNTGFbetaRII) mice] or a combination of the two exposures **(D)** Bar graph showing increased miR-34a expression only with hyperoxia exposure in the antenatal LPS administration (mimicking chorioamnionitis) with/without additional PN hyperoxia exposure in a neonatal rat model. BPD: bronchopulmonary dysplasia; TGFβ: transforming growth factor beta; RA: room air; PN: postnatal; Ang1: angiopoietin 1; Sirt1: sirtuin 1; LPS: lipopolysaccharide. Values are means ± SEM of a minimum of 4 animals in each group. *p<0.05, **p<0.01, ***p<0.001, ****p<0.0001, compared with controls; 1-way ANOVA.

Figure 2

A

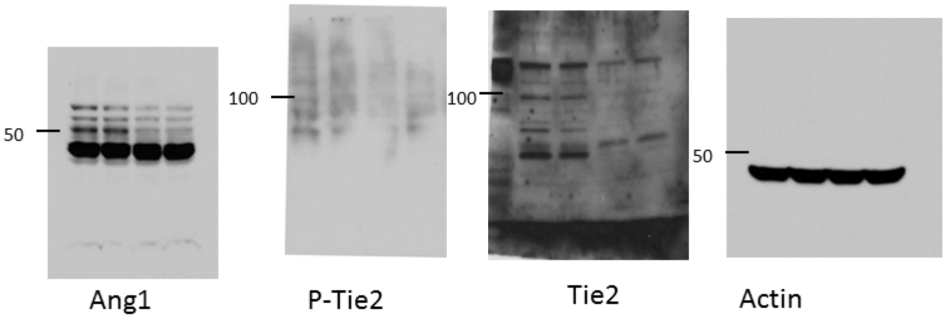

C

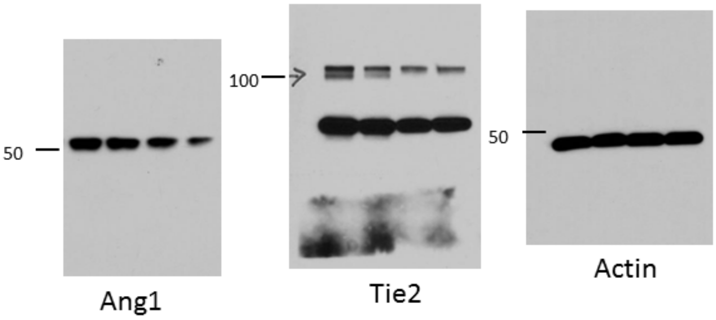

Figure 2

E

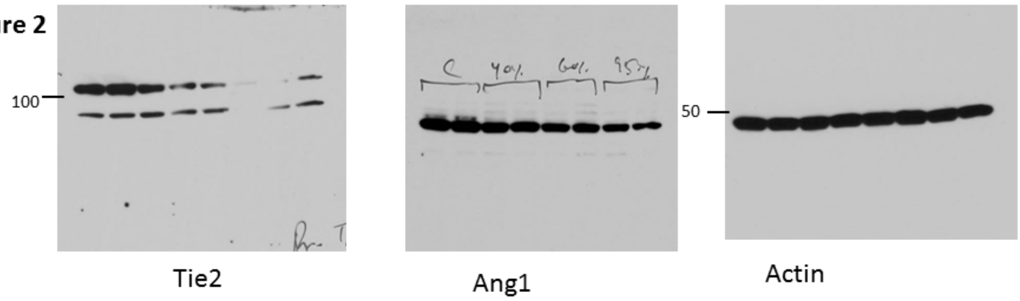

G

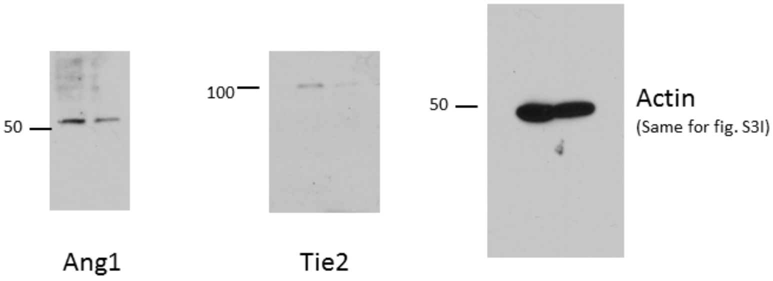

Figure 3

A

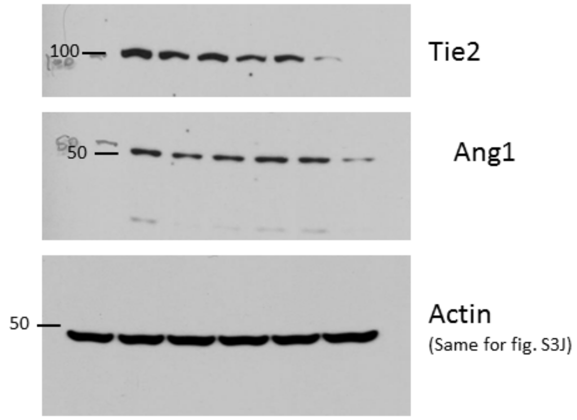

Figure 4

C

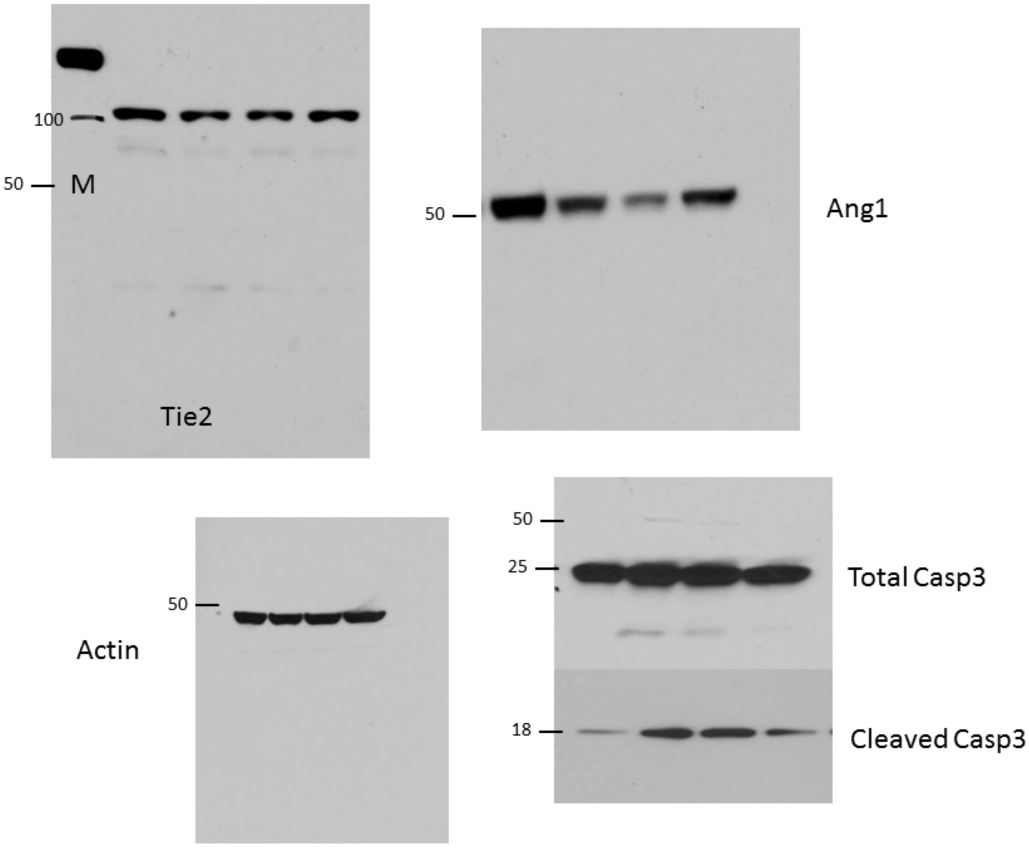

Figure 5

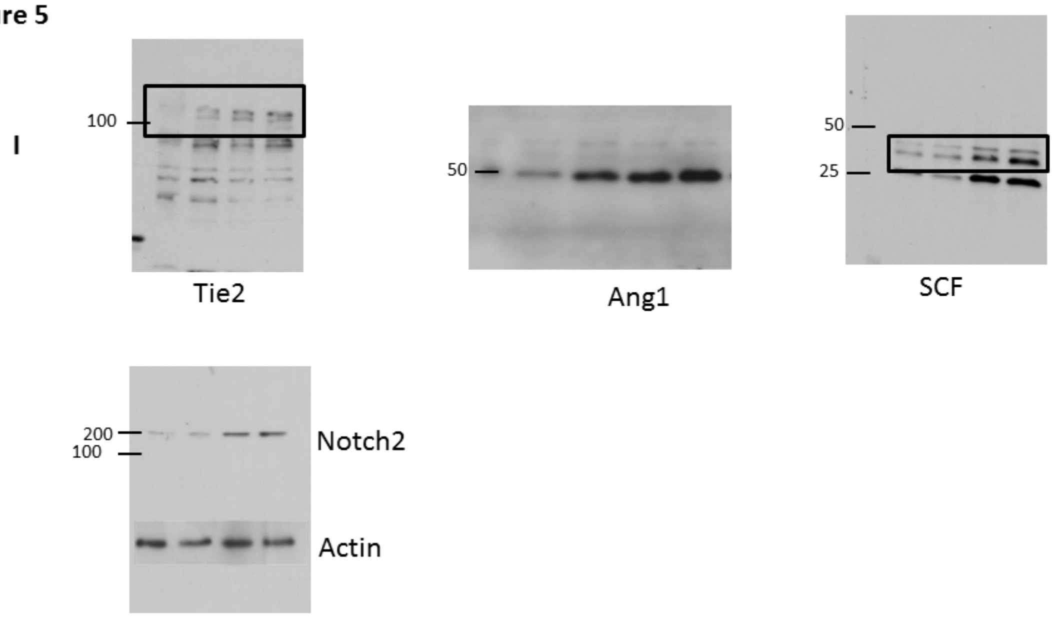

Fig. 5J

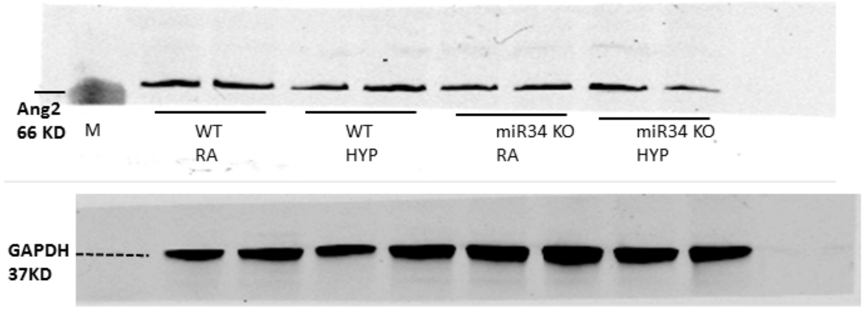

Fig. 5K

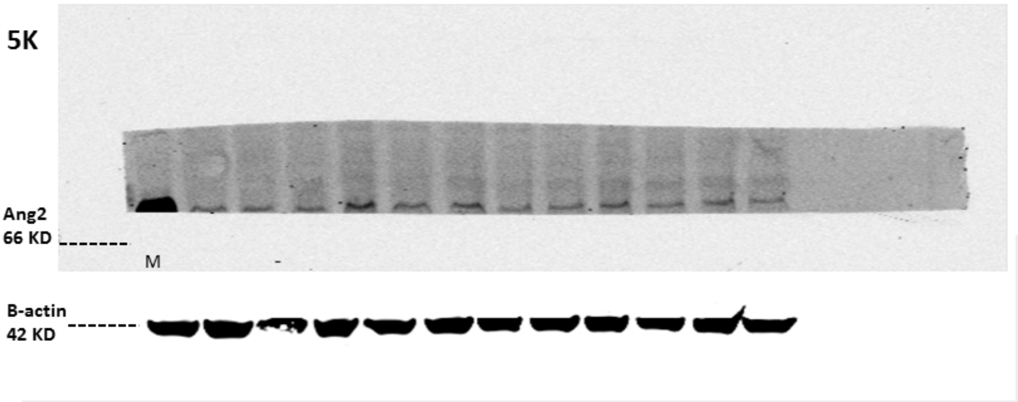

Figure 7

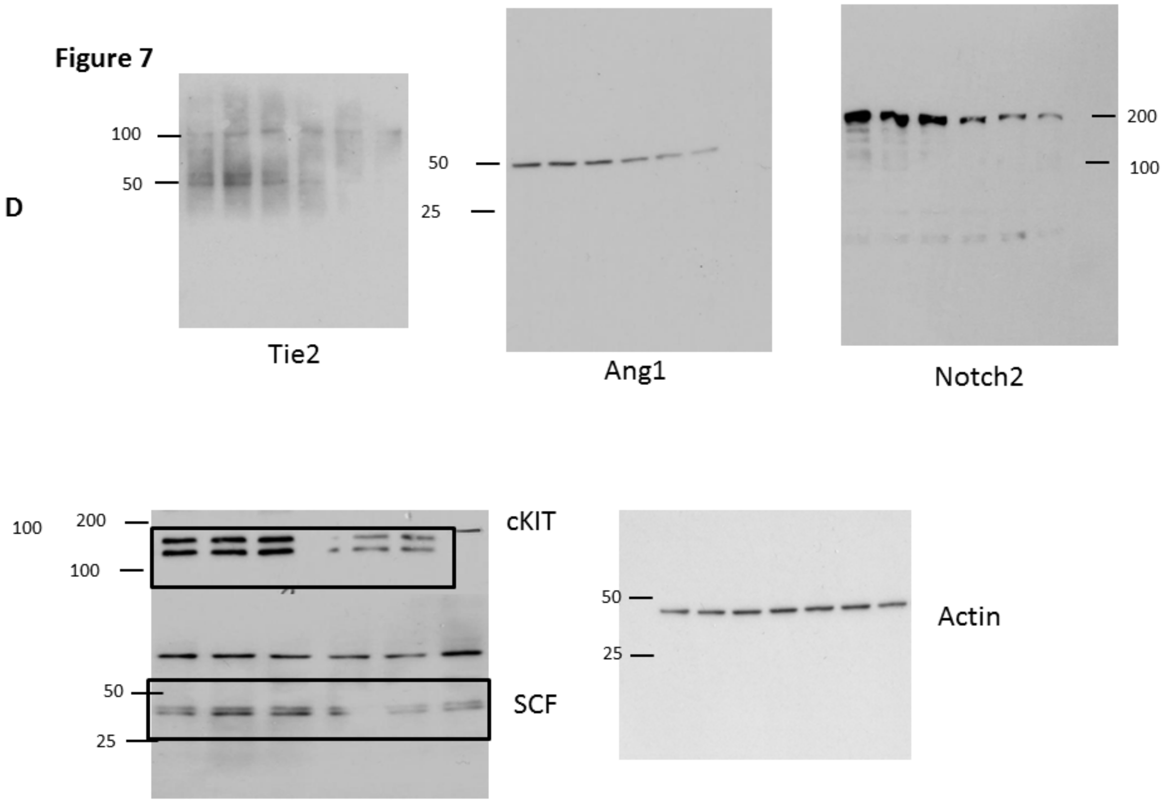
D
100
50
Tie2
50
25
Ang1
200
100
Notch2
100
200
100
cKIT
50
25
SCF
50
25
Actin

Figure 7

E

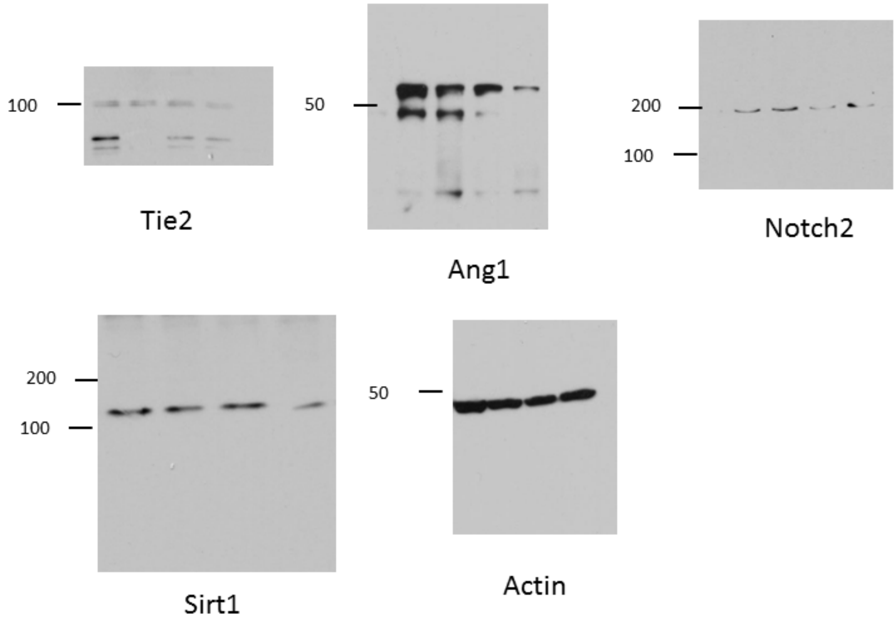

Figure 8

E

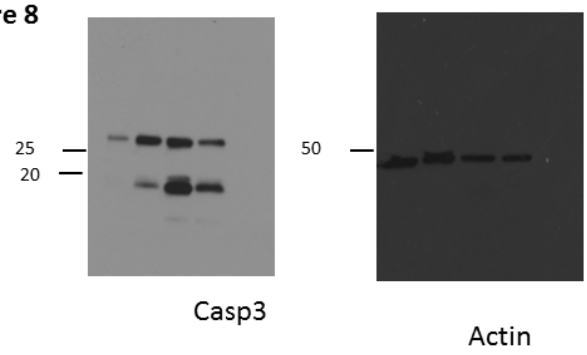

Figure 8

K

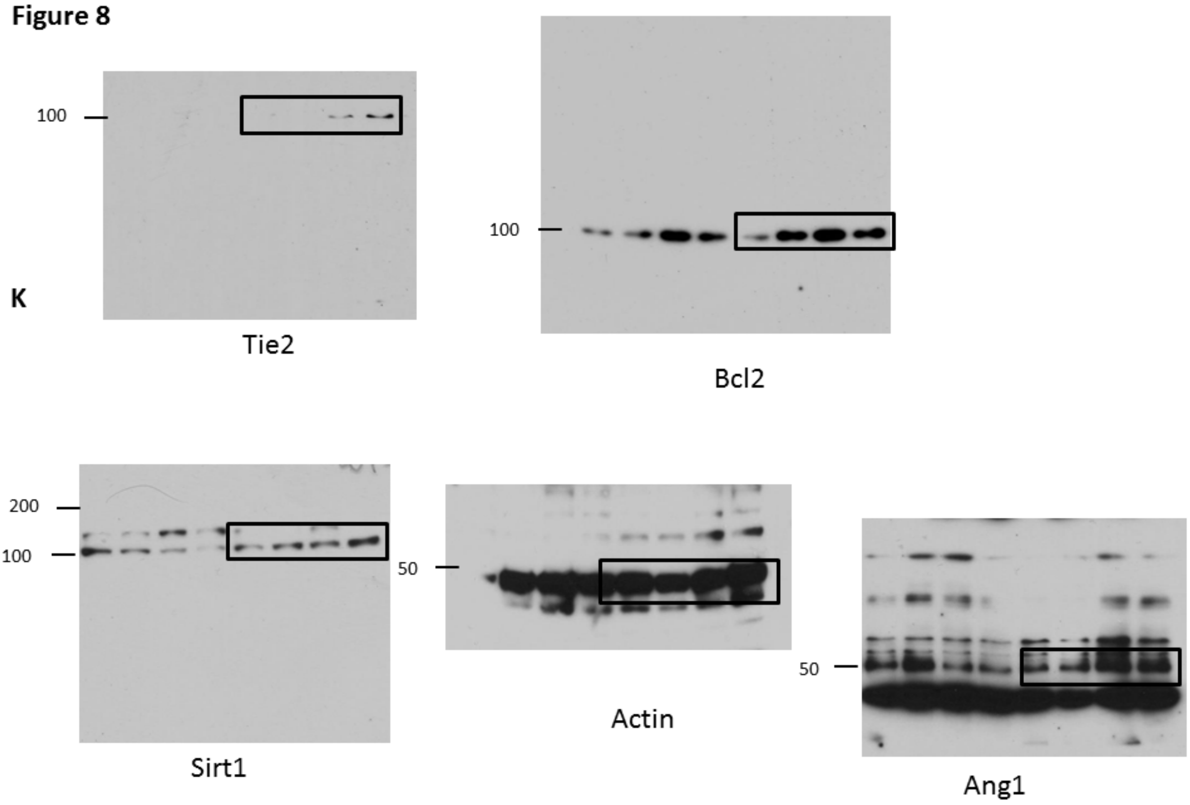

Figure 9

E

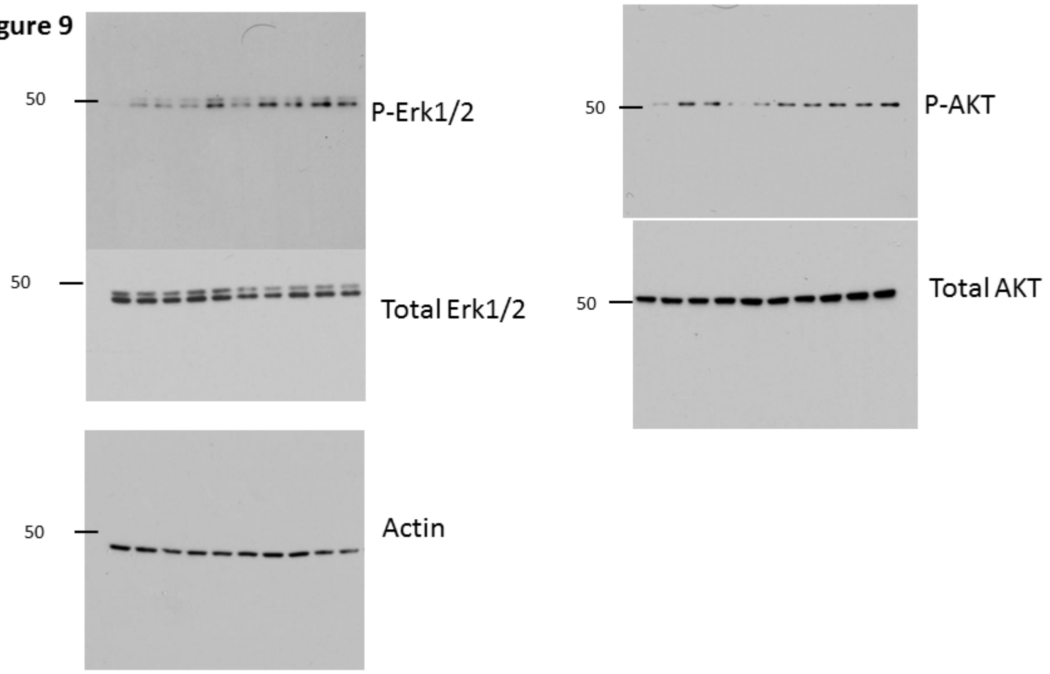

Figure 10

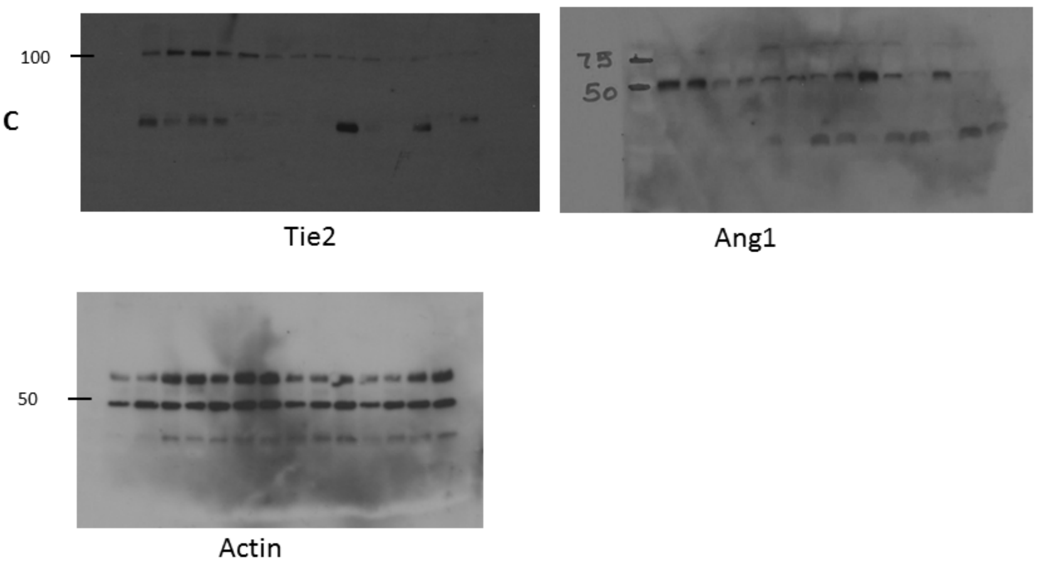

Figure S2

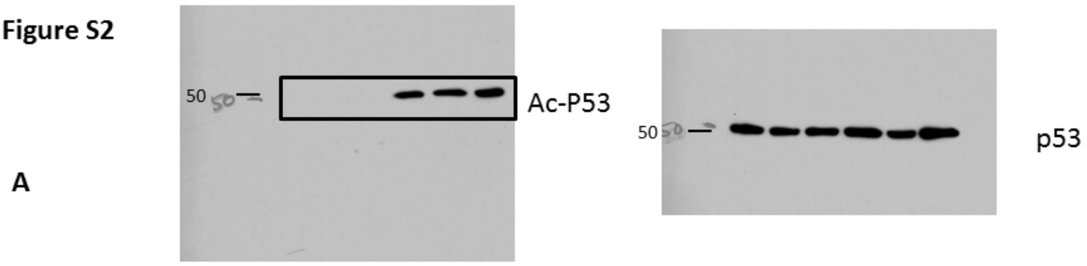

Figure S3

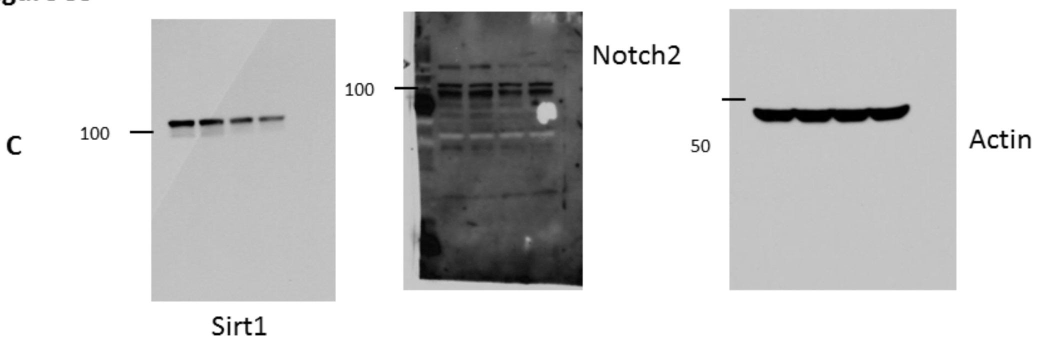

Figure S3

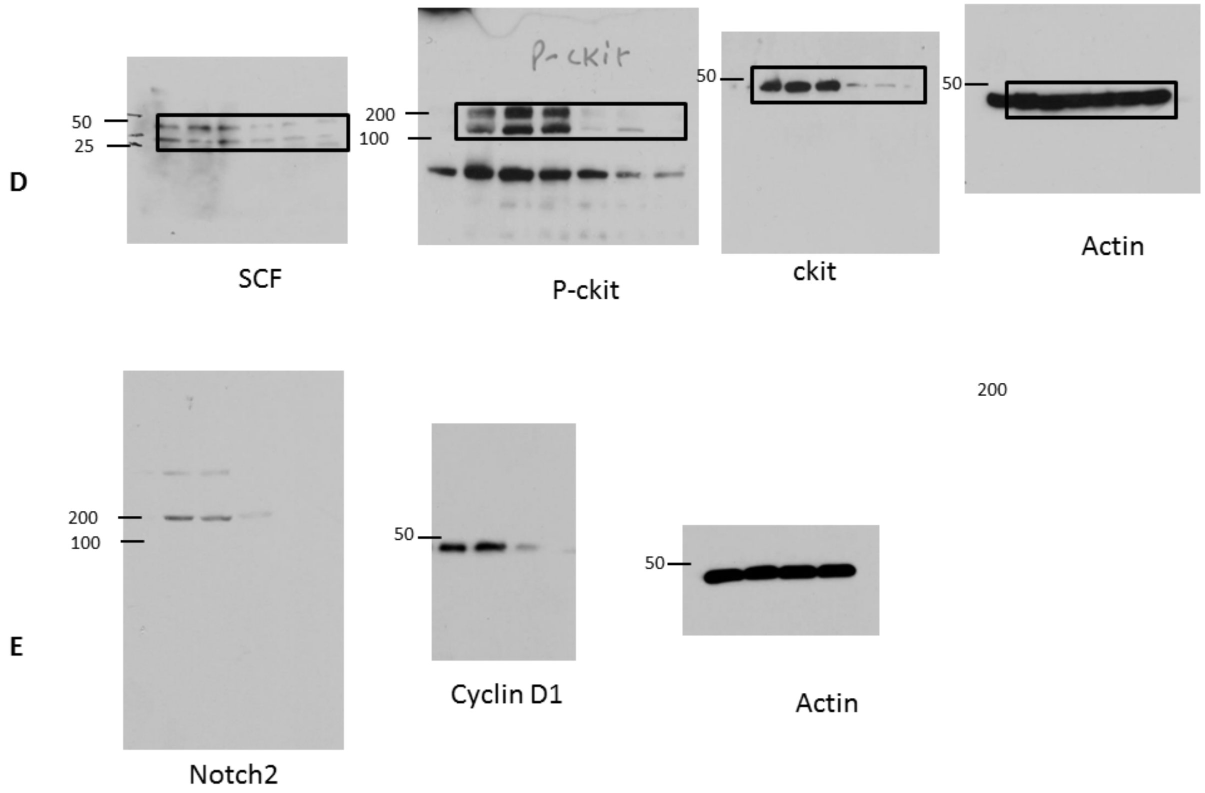

Figure S3

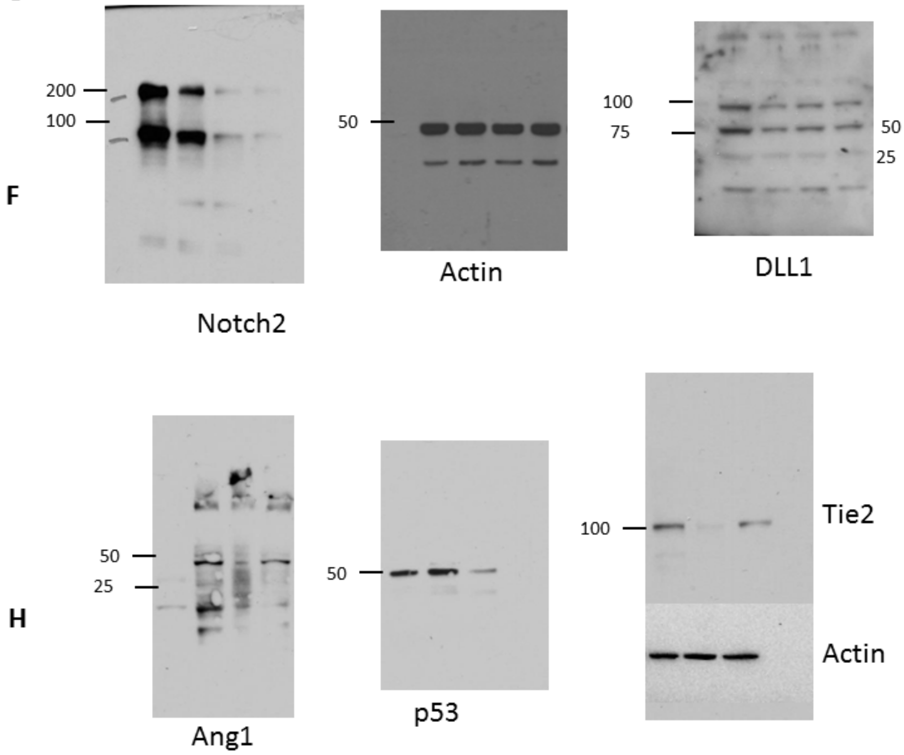

Figure S3

I

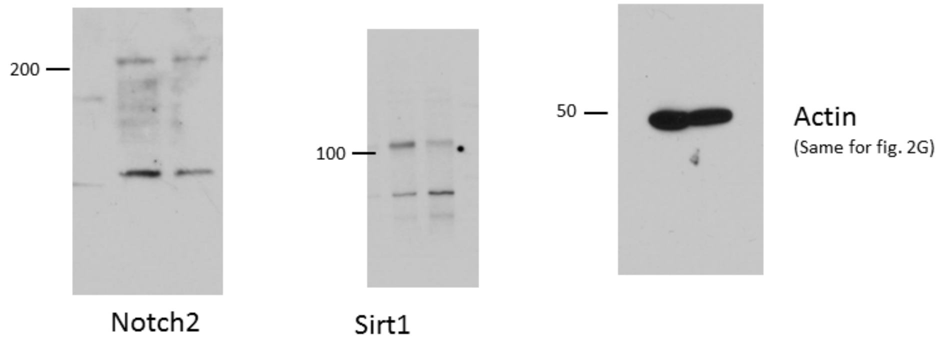
200
100
50
Notch2
Sirt1
Actin
(Same for fig. 2G)

J

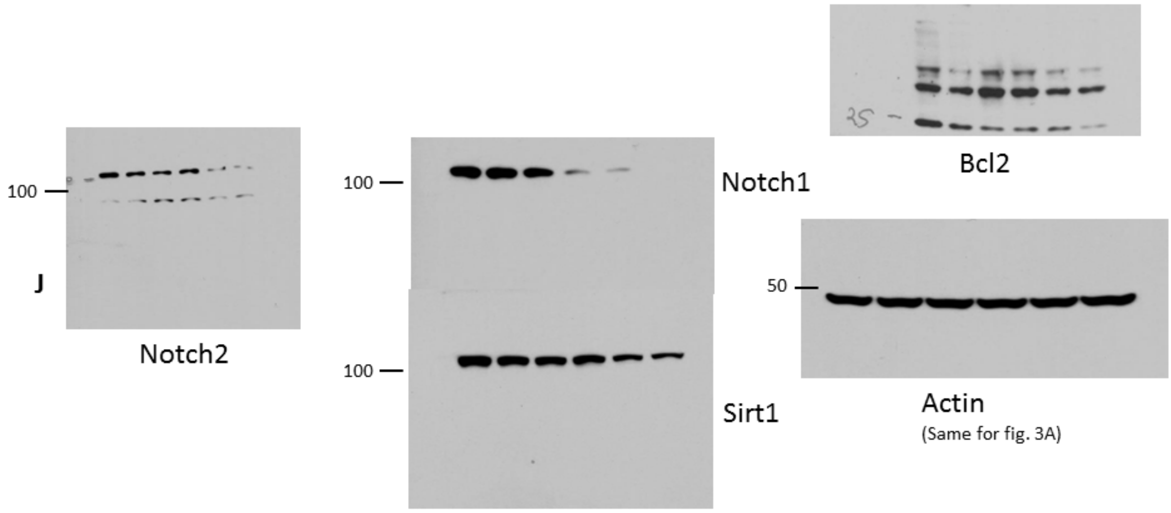
100
100
100
50
25
Notch2
Notch1
Sirt1
Bcl2
Actin
(Same for fig. 3A)

Fig. S8 (top panel)

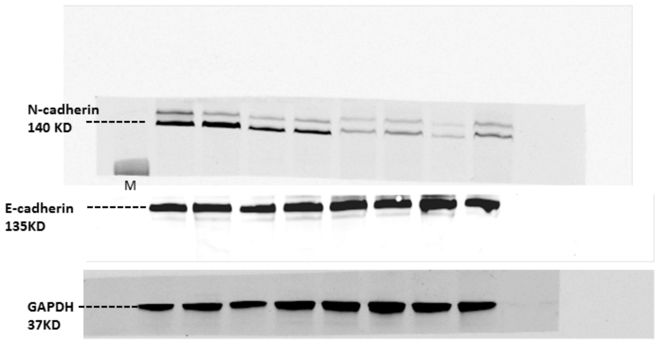

Fig. S8 (bottom panel)

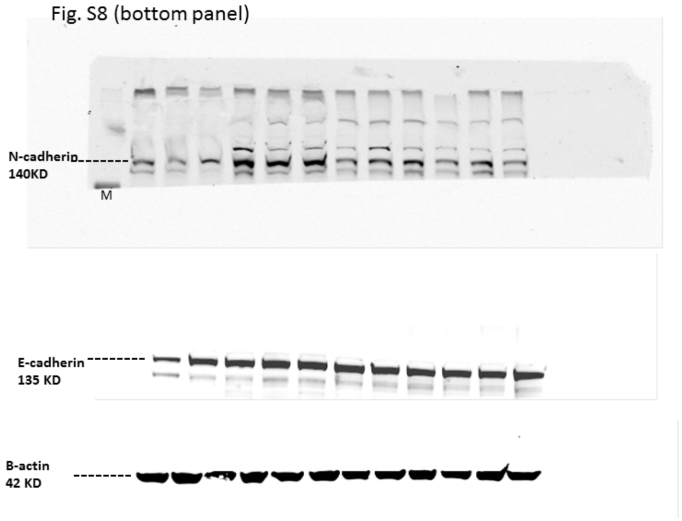

Figure S9

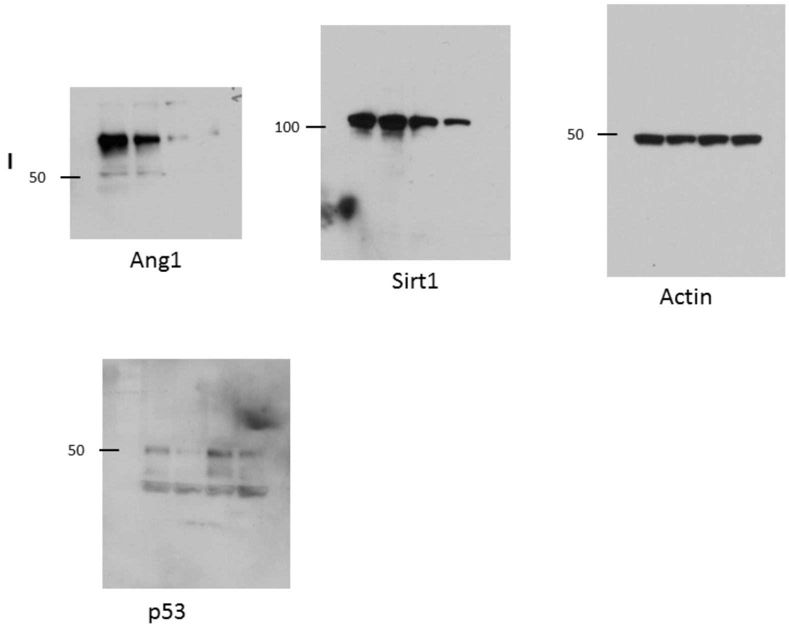

Supplementary Figure 10. Full scans of Western Blots of mentioned Figures. Figures S2, S3, S8 and S9 represent supplementary figures.

Supplementary Table 1: Selected clinical data of infants whose TA cell pellets were used for the *Real time* RT PCR assay for miR-34 expression.

Gestational Age (weeks)	Birth weight (grams)	Age at sample collection (days)	Final Outcome
25	830	1	No BPD
28+1	1038	1	No BPD
25+1	930	2	No BPD
27+1	780	2	No BPD
27	869	1	No BPD
27+6	845	2	BPD
27+1	690	1	BPD
24+6	640	5	BPD
25+2	915	4	BPD
26	976	1	BPD
24+3	670	3	Died
24	552	1	Died
25	745	7	Died
24	603	7	Died
23	550	5	Died

Supplementary Table 2: Selected clinical data of infants whose lungs were used for the *In situ* Hybridization analyses for miR-34 expression.

**Diagnosis**	**Gestational Age (weeks)**	**Birth weight (grams)**	**Age at death (days)**
RDS 1-2d	25+5	500	2
RDS 1-2d	25+0	410	2
RDS 3-7d	30+3	1430	3
RDS 3-7d	26+1	750	4
RDS 3-7d	23+6	700	3
RDS >7d	23+5	610	11
RDS>7d	27+0	540	13
BPD	26+6	890	79
BPD	29	765	125
BPD	29	1070	175
Term	41	4300	3
Term	37	2800	4 hours
